# Serum levels of apoptosis inhibitor of macrophage are associated with hepatic fibrosis in patients with chronic hepatitis C

**DOI:** 10.1186/1471-230X-14-27

**Published:** 2014-02-13

**Authors:** Kumiko Mera, Hirofumi Uto, Seiichi Mawatari, Akio Ido, Yozo Yoshimine, Tsuyoshi Nosaki, Kohei Oda, Kazuaki Tabu, Kotaro Kumagai, Tsutomu Tamai, Akihiro Moriuchi, Makoto Oketani, Yuko Shimada, Masaaki Hidaka, Susumu Eguchi, Hirohito Tsubouchi

**Affiliations:** 1 Digestive and Lifestyle Diseases, Department of Human and Environmental Sciences, Kagoshima University Graduate School of Medical and Dental Sciences, 8-35-1 Sakuragaoka, Kagoshima 890-8544, Japan; 2 Miyazaki Prefectural Industrial Support Foundation, 16500-2 Higashikaminaka, Sadowara-cho, Miyazaki 880-0303, Japan; 3 Department of Surgery, Nagasaki University Graduate School of Biomedical Sciences, 1-7-1 Sakamoto, Nagasaki, 852–8501, Japan; 4 Department of HGF Tissue Repair and Regenerative Medicine, Kagoshima University Graduate School of Medical and Dental Sciences, 8-35-1 Sakuragaoka, Kagoshima 890-8544, Japan

**Keywords:** Apoptosis inhibitor of macrophage, Adipocytokine, Hepatic fibrosis, Hepatitis C virus, Chronic hepatitis, Insulin resistance

## Abstract

**Background:**

Apoptosis inhibitor of macrophage (AIM) and adipocytokines are involved in the metabolic syndrome, which has been putatively associated with the progression of chronic hepatitis C (CHC). However, the association between these cytokines and CHC is not fully elucidated. The aim of this study is to test whether serum levels of AIM and adipocytokines are associated with histological features, homeostasis model assessment-insulin resistance index (HOMA-IR), or whole body insulin sensitivity index (WBISI) in CHC patients.

**Methods:**

Serum samples were obtained from 77 patients with biopsy-proven CHC. In 39 patients without overt diabetes mellitus, a 75 g oral glucose tolerance test (OGTT) was performed and HOMA-IR and WBISI were calculated.

**Results:**

A serum AIM level of ≥1.2 μg/ml was independently associated with advanced hepatic fibrosis (F2 or F3) (odds ratio [OR], 5.612; 95% confidence interval [CI], 1.103–28.563; *P* = 0.038) based on a multivariate analysis, but there was no significant association between AIM and hepatic steatosis or inflammation. Furthermore, a serum leptin level of ≥8.6 ng/ml was independently associated with the presence of hepatic steatosis (≥5%) (OR, 6.195; 95% CI, 1.409–27.240; *P* = 0.016), but not hepatic fibrosis or inflammation. No relationship was observed between levels of adiponectin or resistin and hepatic histological parameters based on a multivariate analysis. Although serum levels of leptin, resistin, and adiponectin were significantly correlated with HOMA-IR and WBISI, there was no significant relationship between serum AIM levels and HOMA-IR or WBISI, respectively.

**Conclusion:**

High serum levels of AIM in CHC patients are potentially related to advanced hepatic fibrosis. AIM and adipocytokines are possibly associated with pathological changes via a different mechanism.

## Background

Chronic hepatitis C virus (HCV) infection is one of the main causes of chronic liver disease. Host factors such as obesity, insulin resistance (IR), and hepatic steatosis have been reported to contribute to the progression from chronic hepatitis C (CHC) to liver cirrhosis and hepatocellular carcinoma (HCC) [1-3]. In addition, chronic HCV infection contributes to the development of hepatic steatosis and IR [4]. Hepatic steatosis and IR have a significant impact on the acceleration of liver injury and hepatic fibrosis in patients with CHC. Furthermore, the pathogenesis of steatosis and IR depend on the viral genotype: increased steatosis has been linked to HCV genotypes 2 and 3 [5,6], and body mass index (BMI) and homeostasis model assessment of IR (HOMA-IR) directly contribute to steatosis in patients infected with HCV genotype 1 [5]. IR is also independently associated with genotypes 1 and 4 [6]. The mechanisms of these interactions, however, are not fully understood.

Apoptosis inhibitor of macrophage (AIM) was initially identified as an apoptosis inhibitor that supports the survival of macrophages against various apoptosis-inducing stimuli [7]. Recently, it has been reported that an increase in the blood levels of AIM is a critical event in the initiation of macrophage recruitment into adipose tissue, which is followed by IR [8]. Miyazaki et al. suggested that AIM is involved in the progression of metabolic syndrome, including obesity and IR, in both an advancing and inhibitory fashion [9], but the impact of AIM on the pathogenesis of HCV-related chronic liver disease has not been investigated.

Adipose tissue, skeletal muscle, and the liver are the major insulin-sensitive organs in the human body. Adipogenesis is the process by which preadipocytes differentiate into adipocytes, which is induced by insulin. Adipose tissue–derived cytokines, so-called adipocytokines, theoretically play an important role in the development of IR [10]. The more familiar adipocytokines include leptin, adiponectin, and resistin. Leptin is a proinflammatory cytokine that accelerates the progression of hepatic fibrosis and exacerbates the inflammatory process in the liver [11]. In contrast, adiponectin reduces hepatic fibrosis and exerts a hepatoprotective effect [12]. Further, resistin, a signaling molecule secreted from adipocytes and monocytes, is known to be involved in inflammatory processes [13], and has recently been reported to be associated with hepatic fibrosis [14,15]. However, their role in HCV-related chronic liver disease is somewhat confusing and the results of various studies have been contradictory [16-18].

In this study, we analyzed the association between serum levels of AIM, leptin, adiponectin, and resistin, and clarified the clinical significance of serum AIM levels in patients with CHC. We then determined whether serum levels of AIM are associated with histological features such as the degree of hepatic steatosis and hepatic inflammation and the stage of hepatic fibrosis. In addition, we determined whether serum levels of AIM and adipocytokines are associated with IR or sensitivity in patients with CHC.

## Methods

### Patients

Seventy-seven consecutive patients with chronic liver disease due to HCV infection admitted to Kagoshima University Medical and Dental Hospital between February 2007 and July 2011 were analyzed in this retrospective study. Ultrasound-guided liver biopsy was performed to confirm the diagnosis. Patients were excluded from this study if they met the following criteria: positive for hepatitis B surface antigen or a history of other types of hepatitis, including autoimmune hepatitis and alcoholic liver disease, or malignancies including HCC. An existence of HCC was confirmed by ultrasonography, computed tomography, or both in patients with chronic hepatitis. Blood samples were obtained just before ultrasound-guided liver biopsy. Informed consent was obtained from all patients. This study was approved by the ethics committees of Kagoshima University Medical and Dental Hospital.

### Laboratory markers

We determined clinical laboratory parameters, such as platelet count, and levels of albumin (Alb), total bilirubin, alanine aminotransferase (ALT), γ-glutamyl transpeptidase (γ-GTP), and α-fetoprotein. Diabetes was defined as fasting plasma glucose ≥126 mg/dL or patients treated with oral hypoglycemic medication or insulin. Hemoglobin A1c (HbA1c) level was calculated as a ratio of HbA1c to the total hemoglobin using chromatography according to the Japan Diabetes Society (JDS) method. Among 60 CHC patients without overt diabetes, including patients treated with oral hypoglycemic medication or insulin, a 75 g oral glucose tolerance test (OGTT) was performed in 39 patients. Serum insulin levels were determined by radioimmunoassay. IR was calculated by the homeostasis model (homeostasis model assessment-insulin resistance, HOMA-IR) using the following formula: HOMA-IR = fasting insulin (μU/ml) × plasma glucose (mg/dl)/405. The whole body insulin sensitivity index (WBISI) was calculated using the following formula: WBISI = 10,000/square root of [fasting glucose × fasting insulin] × [mean glucose × mean insulin during OGTT] [19]. WBISI, which is simple to calculate and provides a reasonable approximation of whole-body insulin sensitivity from OGTT values, is highly correlated with the rate of whole-body glucose disposal during euglycemic insulin clamping [19]. The HCV serotype or genotype was determined with a serological genotyping assay kit (Immunocheck F-HCV Grouping; International Reagents Co., Tokyo, Japan) or the HCV Core Genotype kit (SRL Inc., Tokyo, Japan), respectively. HCV genotype 1b was included with serotype I, and genotypes 2a and 2b were included with serotype II. No other HCV genotypes were detected in this study population. HCV RNA titers were quantified by the Cobas TaqMan PCR assay (Roche, Tokyo, Japan). Patients were considered to have a high viral load if their titers were 5 log IU/mL or higher.

### Serum AIM, adiponectin, leptin, and resistin levels

Serum was obtained by centrifuging the blood sample at 4000 g for 5 min at room temperature and then frozen at -80°C until further use. Serum AIM levels were measured in duplicate using the CircuLex™ Human AIM/CD5L/Spα ELISA kit (CycLex Co., Ltd. Nagoya, Japan). Serum leptin, adiponectin, and resistin levels were measured in duplicate using an enzyme-linked immunoassay (Quantikine ELISA; R&D Systems, Minneapolis, MN).

### Histopathology

Liver biopsy specimens were scored semiquantitatively based on the New Inuyama classification by 3 experienced hepatologists (SM, KT and KO) that were blinded to clinical data [20]. Fibrosis was scored as F0, no fibrosis; F1, portal fibrous widening; F2, portal fibrous widening with bridging fibrosis; F3, bridging fibrosis plus lobular distortion; or F4, probable or definite cirrhosis. Inflammation was graded as A0, none to minimal; A1, mild; A2, moderate; or A3, severe. Hepatic steatosis was evaluated based on the percentage of hepatocytes containing cytoplasmic vacuoles, and the degree of hepatic steatosis was classified as absent (<5%) or present (≥5%), according to a previous study [21].

### Statistical analysis

Results are expressed as means ± standard deviation (SD). *P* values less than 0.05 were considered statistically significant. Statistical analyses were performed using Chi-square or Mann–Whitney U test, as appropriate. Correlation coefficients were calculated by Spearman’s rank correlation analysis. The discriminatory power for each putative marker was described using the receiver operating characteristics (ROC) area under the curve (AUC) (ROC-AUC). Multivariate analyses were performed using logistic regression. Multivariate logistic regression was used to calculate odds ratios (ORs) and 95% confidence intervals (95% CIs) while simultaneously controlling for potential confounders. Cut-off values were obtained from ROC-AUC analysis. Statistical analysis was conducted using PASW Statistics 18 (SPSS Inc., Chicago, IL).

## Results

### Characteristics of patients with biopsy-proven chronic hepatitis

Table 1 summarizes the baseline clinical parameters of all 77 HCV-infected patients who received ultrasound-guided liver biopsy to confirm the diagnosis. The mean age was 56.1 ± 12.4 years (range, 20–76 years) and mean BMI was 23.5 ± 2.9 kg/m^2^ (range, 16.4–32.2 kg/m^2^). Forty-seven patients were infected with serotype I and the remaining 30 patients with serotype II. Complete histological data, including fibrosis stage, inflammation score, and steatosis grade was available for all patients. Fibrosis was absent in 3 patients (3.8%), F1 in 33 (42.9%), F2 in 24 (30.3%), and F3 in 17 (21.5%). As a result, there were no F4 patients among the 77 HCV-infected patients. Hepatic steatosis was present in 18 patients, and absent in 59 patients.

**Table 1:** Baseline clinical, demographic, histologic and metabolic characteristics of 77 patients with chronic hepatitis C

**Patients (number)**	**77**
Age (years)	56.1 ± 12.4
Sex [male/female]	31/46
Body mass index (kg/m^2^)	23.5 ± 2.9
Male (kg/m^2^)	23.8 ± 2.9
Female (kg/m^2^)	23.3 ± 3.1
HCV serotype [I/II]	47/30
Viral load of HCV (log IU/ml)	5.9 ± 1.1
Platelet count (×10^4^/μl)	18.1 ± 7.2
Total cholesterol (mg/dl)	170.7 ± 38.6
Albumin (g/dl)	4.3 ± 0.4
ALT (IU/l)	64.5 ± 53.9
γ-GTP (IU/l)	50.1 ± 62.2
Diabetes [absent/present]	60/17
Hemoglobin A1c (%)	5.4 ± 0.6 (n = 75)
Fasting plasma glucose (mg/dl)	97.0 ± 22.1
Fasting insulin (μU/ml)	9.8 ± 5.7 (n = 40)
HOMA-IR	2.2 ± 1.5 (n = 40)
Leptin (ng/ml)	7.6 ± 5.1
Adiponectin (ng/ml)	9.0 ± 6.2
Resistin (ng/ml)	8.5 ± 4.6
AIM (μg/ml)	1.39 ± 0.86
Hyaluronic acid (ng/ml)	95.9 ± 114.0 (n = 75)
α-fetoprotein (ng/ml)	7.1 ± 11.2
Histologic characteristics	
Activity [A0-1/2-3]	32/45
Fibrosis [F0-1/2-3]	36/41
Steatosis [<5%/≥5%]	59/18

Data are shown as the means ± standard deviations or number.

HCV, hepatitis C virus; ALT, alanine aminotransferase; γ-GTP, γ-glutamyl transpeptidase; HOMA-IR, homeostasis model assessment-insulin resistance; AIM, apoptosis inhibitor of macrophage.

### Association between serum AIM levels and other laboratory data in patients with chronic hepatitis C

Among the laboratory parameters listed in Table 2, serum AIM levels were positively correlated with ALT and hyaluronic acid. Serum AIM levels were negatively correlated with platelet count, total cholesterol, and albumin, suggesting that serum AIM levels are potentially associated with hepatic fibrosis and hepatic reserve.

**Table 2:** Association between serum levels of apoptosis inhibitor of macrophage and clinical parameters in patients with chronic hepatitis C

**Factor**	**Number**	**Correlation coefficient**	***P *** value*
Age	77	0.085	0.463
Body mass index	77	-0.074	0.521
Viral load of HCV	77	-0.152	0.188
Platelet count	77	-0.374	0.001
Total cholesterol	77	-0.325	0.004
Albumin	77	-0.309	0.006
ALT	77	0.253	0.027
γ-GTP	77	0.222	0.052
Hemoglobin A1c	75	-0.069	0.559
Fasting plasma glucose	77	0.075	0.517
Leptin	77	-0.039	0.740
Adiponectin	77	0.029	0.800
Resistin	77	0.034	0.771
Hyaluronic acid	75	0.330	0.004
α-fetoprotein	77	0.008	0.942

*Calculated by Spearman’s rank correlation analysis.

ALT, alanine aminotransferase; γGTP, γ-glutamyl transpeptidase; HOMA-IR, homeostasis model assessment-insulin resistance.

### Diagnostic value of serum AIM levels for advanced hepatic fibrosis

Platelet count and serum levels of hyaluronic acid are known diagnostic markers of hepatic fibrosis. To evaluate the ability of AIM to diagnose advanced hepatic fibrosis (F2–3), we examined AIM in addition to hepatic fibrosis markers. In an AUC-ROC analysis, hyaluronic acid was the strongest diagnostic marker for advanced fibrosis (AUC-ROC, 0.854), and the AUC-ROCs for platelet count and AIM were 0.769 and 0.764, respectively. In addition, AUC-ROC analysis revealed that a serum AIM level of 1.2 μg/ml was the optimal cut-off value to differentiate between absent or mild hepatic fibrosis (F0–1) and advanced hepatic fibrosis, with 73.2% sensitivity and 75.0% specificity.

### Factors associated with the severity of hepatic fibrosis

Table 3 shows the demographic, clinical, and biochemical characteristics of patients with advanced fibrosis (F2–3) versus absent or mild fibrosis (F0–1). Univariate analysis revealed that age, platelet count, albumin, ALT, γ-GTP, AIM, and hyaluronic acid were significantly associated with advanced hepatic fibrosis. In contrast, serum leptin, adiponectin, and resistin were not associated with advanced hepatic fibrosis. In a multivariate regression model including age (≥55 years), platelet count (<17 × 10^4^/μl), albumin (<4.4 g/dl), ALT (≥43 IU/l), γ-GTP (≥27 IU/l), AIM (≥1.2 μg/ml), and hyaluronic acid (≥40 ng/ml), advanced hepatic fibrosis was independently associated with ALT, AIM, and hyaluronic acid (Table 4).

**Table 3:** Comparison of the clinical characteristics of chronic hepatitis C patients with (F2–3) or without (F0–1) advanced hepatic fibrosis

		**F0 – F1**			**F2 – F3**		
		**(n = 36)**			**(n = 41)**		
**Factor**	**Mean or number**	**Maximum**	**Minimum**	**Mean or number**	**Maximum**	**Minimum**	***P *** value^*^
Age (years)	53	71	22	59	76	20	0.029
Sex (male/female)	14/22			17/24			0.818
Body mass index (kg/m^2^)	23.2	30.1	16.4	23.6	32.2	17.8	0.814
HCV serotype (I/II)	21/15			26/15			0.815
Viral load (log IU/ml)	6	7.3	2.7	5.8	7.3	2.4	0.606
Platelet count (×10^4^/μl)	21	41.9	9.8	15.5	39.7	5.4	<0.001
Total cholesterol (mg/dl)	179.4	295	84	163	251	92	0.071
Albumin (g/dl)	4.5	5.1	3.5	4.1	4.9	2.9	<0.001
ALT (IU/L)	40.3	181	13	85.8	244	17	<0.001
γ-GTP (IU/L)	31.7	114	12	66.3	468	12	0.001
Diabetes (absent/present)	6/30			11/30			0.283
Fasting plasma glucose (mg/dl)	95.3	155	75	98.6	196	72	0.810
Hemoglobin A1c (%)	5.3 (n = 35)	6.7	4.6	5.5 (n = 40)	7.6	4.6	0.861
Leptin (ng/ml)	6.9	21.8	1.0	8.2	21.3	1.1	0.086
Adiponectin (ng/ml)	8.8	27.3	1.7	9.1	26.7	1.1	0.653
Resistin (ng/ml)	8.1	27.6	2.5	8.9	17.8	3	0.156
AIM (μg/ml)	1.06	2.02	0.41	1.68	6.44	0.76	<0.001
Hyaluronic acid (ng/ml)	42.6	10.0	292.7	145.0 (n = 39)	10.0	636.0	<0.001

*Calculated by Chi-square test for categorical variables or Mann–Whitney U test for continuous variables.

ALT, alanine aminotransferase; γ-GTP, γ-glutamyl transpeptidase; HOMA-IR, homeostasis model assessment-insulin resistance; AIM, apoptosis inhibitor of macrophage.

**Table 4:** Multiple logistic regression analysis for factors associated with advanced hepatic fibrosis in patients with chronic hepatitis C

**Variable**	**Odds ratio**	**95% confidential interval**	***P *** **value**
Age (≥55 years)	1.025	0.168-6.253	0.979
Platelet count (<17 × 10^4^/μl)	5.617	0.933-33.826	0.060
Albumin (<4.4 g/dl)	2.236	0.470-10.638	0.312
ALT (≥43 IU/l)	5.906	1.179-29.589	0.031
γ-GTP (≥27 IU/l)	2.447	0.489-12.247	0.276
AIM (≥1.2 μg/ml)	5.612	1.103-28.563	0.038
Hyaluronic acid (≥40 ng/ml)	11.617	2.233-60.443	0.004

ALT, alanine aminotransferase; γ-GTP, γ-glutamyl transpeptidase; AIM, apoptosis inhibitor of macrophage.

### Factors associated with the severity of hepatic steatosis and inflammation

The relationship between serum levels of AIM or adipokines and the severity of histological features including hepatic steatosis and inflammation were assessed in CHC patients. Serum albumin, γ-GTP, leptin, resistin, and hyaluronic acid, as well as the presence of diabetes, were associated with hepatic steatosis (≥5%) in the univariate analysis (Table 5). Based on multivariate regression analysis that included albumin (≥4.0 g/dl), γ-GTP (≥44 IU/L), leptin (≥8.6 ng/ml), resistin (≥8.8 ng/ml), hyaluronic acid (≥76.4 ng/ml), presence of diabetes, and age (≥55 years), leptin was independently associated with hepatic steatosis (Table 6). Furthermore, in the univariate analysis, age, platelet count, and serum levels of albumin, ALT, γ-GTP, AIM, and hyaluronic acid were associated with more severe hepatic inflammation (A2–3, Table 7), but there were no statistically significant association between these parameters and hepatic inflammation in a multivariate regression model including age (≥55 years), platelet count (<17 × 10^4^/μl), albumin (<4.4 g/dl), ALT (≥43 IU/L), γ-GTP (≥27 IU/L), AIM (≥1.2 μg/ml), and hyaluronic acid (≥45.4 ng/ml) (all *P* >0.1 in multivariate analysis).

**Table 5:** Comparison of the clinical characteristics of chronic hepatitis C patients with or without hepatic steatosis (≥5%)

		**Steatosis (<5%)**			**Steatosis (≥5%)**		
		**n = 59**			**n = 18**		
**Factor**	**Mean or number**	**Maximum**	**Minimum**	**Mean or number**	**Maximum**	**Minimum**	***P *** value^*^
Age (years)	55.2	76	20	59.1	69	33	0.257
Sex (male/female)	26/33			5/13			0.217
Body mass index (kg/m^2^)	22.3	30.1	16.4	23.8	32.2	19.9	0.800
Platelet count (×10^4^/μl)	18.3	41.9	7.4	17.1	36.2	5.4	0.434
Total cholesterol (mg/dl)	171.4	295	84	168.4	222	114	0.814
Albumin (g/dl)	4.3	5.1	3.3	4.0	4.9	2.9	0.023
ALT (IU/l)	63.1	244	13	69.1	191	20	0.125
γ-GTP (IU/l)	40.6	239	12	81.4	468	12	0.010
Diabetes (absent/present)	50/9			10/8			0.009
Fasting plasma glucose (mg/dl)	95.9	196	72	100.8	150	80	0.145
Hemoglobin A1c (%)	5.3 (n = 57)	7.6	4.6	5.6	7.1	4.7	0.084
Leptin (ng/ml)	7.0	21.8	1.0	9.6	17.3	3.5	0.006
Adiponectin (ng/ml)	9.3	27.3	1.1	8	26.1	1.7	0.493
Resistin (ng/ml)	8.1	27.6	2.5	9.9	17.5	4	0.042
AIM (μg/ml)	1.37	4.56	0.41	1.43	6.44	0.72	0.290
Hyaluronic acid (ng/ml)	71.9 (n = 57)	292.7	10	171.6	636.0	11.2	0.021

*Calculated by Chi-square test for categorical variables or Mann–Whitney U test for continuous variables.

ALT, alanine aminotransferase; γGTP, γ-glutamyl transpeptidase; HOMA-IR, homeostasis model assessment-insulin resistance; AIM, apoptosis inhibitor of macrophage.

**Table 6:** Multiple logistic regression analysis for factors associated with hepatic steatosis (≥5%) in patients with chronic hepatitis C

**Variable**	**Odds ratio**	**95% confidential interval**	***P *** **value**
Age (≥55 years)	4.839	0.788-29.733	0.089
Albumin (<4.0 g/dl)	1.771	0.310-10.099	0.520
γ-GTP (≥44 IU/L)	3.077	0.741-12.783	0.122
Diabetes mellitus (present)	2.853	0.611-13.331	0.182
Leptin (≥8.6 ng/ml)	6.195	1.409-27.240	0.016
Resistin (≥8.8 ng/ml)	3.396	0.823-14.006	0.091
Hyaluronic acid (≥76.4 ng/ml)	1.814	0.424-7.766	0.422

γ-GTP, γ-glutamyl transpeptidase.

**Table 7:** Comparison of the clinical characteristics of chronic hepatitis C patients with (A2–3) or without (A0–1) severe hepatic inflammation

		**A0-A1**		**A2-A3**			
		**n = 32**		**n = 45**			
**Factor**	**Mean or number**	**Maximum**	**Minimum**	**Mean or number**	**Maximum**	**Minimum**	***P *** value^*^
Age (years)	51.9	70	20	59.1	76	24	0.039
Sex (male/female)	14/18			17/28			0.598
Body mass index (kg/m^2^)	23.1	30.1	16.4	23.7	32.2	17.8	0.694
Platelets (×10^4^/μl)	21.3	41.9	11.1	15.8	39.7	5.4	<0.001
Total cholesterol (mg/dl)	180.9	295	101	163.4	251	84	0.122
Albumin (g/dl)	4.4	5.1	3.3	4.2	4.9	2.9	0.003
ALT (IU/l)	42.2	165	13	80.3	244	14	0.003
γ-GTP (IU/l)	32.3	114	12	62.8	468	12	0.006
Diabetes (absent/present)	6/26			11/34			0.553
Fasting plasma glucose (mg/dl)	97.8	196	75	96.5	176	72	0.983
Hemoglobin A1c (%)	5.4 (n = 31)	7.6	4.6	5.4 (n = 44)	7.1	4.7	0.643
Leptin (ng/ml)	7.2	21.8	1	7.8	20.7	1.1	0.247
Adiponectin (ng/ml)	8.9	27.3	1.1	9	26.7	1.7	0.463
Resistin (ng/ml)	8.6	27.6	2.5	8.5	19.7	3	0.687
AIM (μg/ml)	1.17	4.56	0.41	1.54	6.44	0.76	0.001
Hyaluronic acid (ng/ml)	58.8	10.0	292.7	123.4 (n = 43)	12.1	636.0	0.001

*Calculated by Chi-square test for categorical variables or Mann–Whitney U test for continuous variables.

ALT, alanine aminotransferase; γGTP, γ-glutamyl transpeptidase; HOMA-IR, homeostasis model assessment-insulin resistance; AIM, apoptosis inhibitor of macrophage.

### Association between serum levels of AIM and IR in patients with chronic hepatitis C

**Figure 1:**
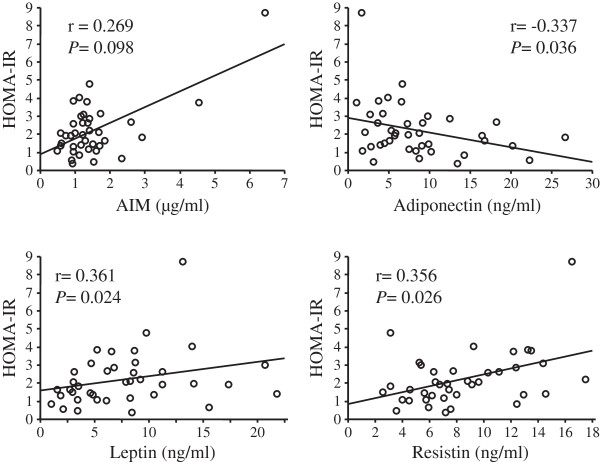
**Correlation between serum levels of AIM, adiponectin, leptin, or resistin and HOMA-IR.** Resistin and leptin levels were positively and serum adiponectin levels were negatively correlated with HOMA-IR. There was a tendency towards a positive correlation between serum AIM levels and HOMA-IR. AIM, apoptosis inhibitor of macrophage; HOMA-IR, homeostasis model assessment-insulin resistance. Correlation coefficients were calculated by Spearman’s rank correlation analysis.

**Figure 2:**
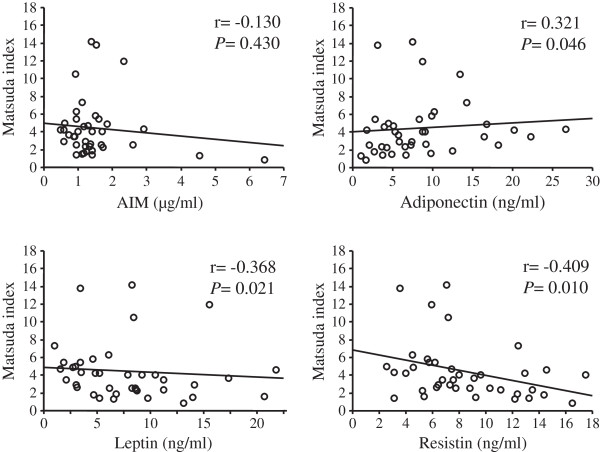
**Correlation between serum levels of AIM, adiponectin, leptin, or resistin and WBISI.** Serum AIM levels were not correlated with WBISI, but serum adiponectin levels were positively and serum resistin and leptin levels were negatively correlated with WBISI. AIM, apoptosis inhibitor of macrophage; WBISI, whole body insulin sensitivity index [19]. Correlation coefficients were calculated by Spearman’s rank correlation analysis.

Among 77 CHC patients who underwent liver biopsy, 39 patients without overt diabetes mellitus were available for the OGTT. In these 39 patients, there was no significant association between serum levels of AIM and adiponectin, leptin, or resistin. Serum levels of AIM tended to be correlated with HOMA-IR, but this association did not show statistical significance (Figure 1, r = 0.269, *P* = 0.098). Furthermore, serum levels of leptin and resistin were positively correlated with HOMA-IR, and serum levels of adiponectin were negatively correlated with HOMA-IR (Figure 1). In addition, serum levels of adiponectin, leptin, and resistin were significantly associated with WBISI, but serum levels of AIM were not correlated with WBISI (Figure 2, r = 0.130, *P* = 0.430).

## Discussion

HCV infection is the most important cause of chronic hepatitis, liver cirrhosis, and HCC. The incidence of HCC increases with increasing severity of hepatic fibrosis in patients with HCV infection. Hepatic inflammation and steatosis are thought to affect the progression of hepatic fibrosis. In addition, IR and obesity are thought to be associated with histopathology of the liver. Several adipocytokines and macrophage-derived molecules are reported to be associated with IR and obesity; we examined the association between these molecules and histopathological features in the liver. In this study, we demonstrated that serum AIM levels in patients with CHC were positively associated with hepatic fibrosis, but leptin, adiponectin, or resistin levels did not show this association. In contrast, serum levels of adiponectin, leptin and resistin were associated with IR, but AIM was not. Although the pathophysiological mechanism of AIM in CHC patients remains unclear, our study is the first to illustrate the clinical significance of AIM in patients with CHC.

In this study, we showed that serum AIM levels as determined by ELISA were higher in CHC patients with severe hepatic fibrosis compared to those with no or mild hepatic fibrosis. In addition, we confirmed that six HCV-infected cirrhotic patients who underwent liver transplantation had serum AIM levels greater than 1.2 μg/ml (data not shown). Gangadharan et al. previously reported that serum levels of the AIM protein were elevated in hepatitis C patients with liver cirrhosis compared to healthy controls, using a proteomics method based on 2-dimensional gel electrophoresis (2-DE) [22]. They speculated that the increased serum AIM levels observed in cirrhotic patients may be associated with HCV infection rather than cirrhosis. However, serum AIM levels determined by proteomics using 2-DE analysis were previously reported to reflect the severity of hepatic fibrosis in nonalcoholic fatty liver disease [23]. ALT ≥43 IU/l was more strongly associated with advanced hepatic fibrosis compared to AIM levels ≥1.2 μg/ml in our study; this may suggest that any association is indeed weak, but it is well known that ALT levels in patients with liver cirrhosis are lower than levels in patients with chronic hepatitis. Further, ALT <43 IU/L was observed in half of the patients with HCV-associated liver cirrhosis who received liver transplantation (data not shown). Therefore, the serum concentration of AIM is potentially a marker of hepatic fibrosis in chronic liver disease. Further validation studies involving patients with chronic liver disease, including those with HCV-associated liver cirrhosis, chronic hepatitis B, autoimmune liver disease, and fatty liver disease, are needed.

AIM is a member of the scavenger receptor cysteine-rich superfamily that was initially identified as an inhibitor of apoptosis that supports the survival of macrophages against different types of pro-apoptotic stimuli [5]. It is reportedly solely produced by tissue macrophages, including Kupffer cells [24,25]. In addition, we observed that mRNA expression of AIM in the human hepatic stellate cell line LI90 was weak, but it was strong in the human macrophage cell line THP-1 (data not shown). Which cell types express AIM in chronic liver disease was not determined in this study; Kupffer cells should be the main source of AIM in liver with hepatic fibrosis. Kupffer cells are resident macrophages of the liver. Activated Kupffer cells play a pivotal role in triggering and maintaining inflammation in chronic liver disease including CHC and nonalcoholic steatohepatitis (NASH) [26,27]. Persistent hepatic inflammation and hepatic stellate cell resistance to apoptosis are some of the mechanisms involved in progressive hepatic fibrogenesis [28]. AIM induced by Kupffer cells may contribute to these mechanisms, and we speculate that elevated serum AIM levels may be due to increased production in vivo. However, our study was cross-sectional, and a longitudinal study involving a larger number of patients with an even distribution among the different stages of fibrosis is needed to elucidate the causal relationship between AIM and hepatic fibrosis. In addition, the association between serum AIM levels and hepatic reserve or renal function should be closely examined.

IR is independently associated with the severity of fibrosis in chronic liver disease, including CHC and NASH [29,30]. In addition, patients may have advanced hepatic fibrosis complicated by IR, abnormal glucose metabolism, or diabetes mellitus [31]. IR is thought to directly activate profibrogenic signaling pathways [32,33]. However, the molecular mechanisms through which IR influences hepatic fibrosis are not fully elucidated. Recently, it was reported that the whole-body glucose intolerance and IR observed in obese AIM wild-type mice were less severe in obese AIM knockout mice, as shown by intraperitoneal glucose and insulin tolerance tests [8,9]. In our study, serum levels of AIM were tended to be correlated with HOMA-IR, although this correlation was not significant and AIM levels were not associated with the Matsuda index of whole-body insulin sensitivity [19]. Simple indices based on fasting levels of glucose and insulin (e.g. HOMA-IR) assesses hepatic IR more than peripheral insulin sensitivity [34]. Although it is unclear whether serum levels of AIM affect hepatic IR, high serum levels of AIM associated with hepatic fibrosis potentially connect hepatic fibrosis to IR. Further examination in patients with the same stage of fibrosis is needed.

In this study, serum levels of leptin were associated with HOMA-IR and hepatic steatosis, but not hepatic fibrosis and inflammation. Previous studies similarly showed that serum leptin levels are associated with IR [18] and hepatic steatosis [35]. In contrast, Cua et al. also reported that serum leptin levels are not associated with histological findings [18], but Piche et al. showed an association between serum leptin levels and the severity of hepatic fibrosis [36]. Resistin levels were reported to be inversely associated with hepatic fibrosis and resistin may stimulate fibrogenesis directly or indirectly through hepatic inflammation [14,16], but our study indicates that serum levels of resistin are not associated with hepatic fibrosis. In addition, Jonsson et al. reported that serum adiponectin levels were correlated with HOMA-IR and inversely correlated with steatosis in HCV-infected male subjects [37]. However, Liu et al. reported that adiponectin levels do not correlate with histological features such as hepatic steatosis, although low adiponectin levels are associated with HOMA-IR in patients with CHC [38]. Thus, prior studies on the role of the adipocytokines including adiponectin, leptin, and resistin, in the pathogenesis of histological changes in the liver and IR in patients with CHC have yielded conflicting results [16]. It is noteworthy that an association between BMI and steatosis has been reported [5,6]. Further, more severe steatosis is associated with more rapid progression of fibrosis [39]. In our study population, BMI is lower and hepatic steatosis is less severe compared to a previous study [21]. In addition to BMI and histological findings, many other factors such as study population and sample size should be carefully considered when interpreting the data.

There are several limitations to this study. First, there were no F4 patients among the 77 HCV-infected patients analyzed in whom ultrasound-guided liver biopsy was performed. This selection bias was likely due to the fact that patients with suspected liver cirrhosis usually do not undergo liver biopsy for histological examination. In addition, the number of patients with OGTT data was small (only 39 patients). Further examination using a large number of patients with OGTT data available, including patients with compensated cirrhosis, is needed. Second, we employed a 5% cut-off for the amount of fat within the liver; some reproducible histological scales use the categories of mild (5–10%), moderate (11–30%), and severe steatosis (>30%) [39]. Itoh et al. also divided patients into groups with 0–10% and >10% hepatic steatosis [40]. However, the proportion of patients with ≥5% or ≥11% hepatic steatosis in our study population was small (18/77 [23.4%] or 3/77 [3.9%], respectively) compared to earlier studies. The reason for this difference in the distribution of hepatic steatosis severity is unclear, but there might be regional differences. In addition, the cut-off of 5% steatosis may not be clinically significant, and we should keep in mind that factors associated with steatosis might depend on the cut-off value used to define steatosis. Thus, the association between serum AIM levels and hepatic steatosis should be re-evaluated in studies with more subjects that include patients with severe hepatic steatosis. Third, several confounders such as alcohol consumption, previous interferon treatment, HCV genotype, and genetic factors including interleukin 28B polymorphism [41], were not considered. The association between examined cytokines and hepatic fibrosis or steatosis in patients with CHC should be further examined in large-scale nationwide studies in the future.

## Conclusions

Serum AIM levels in CHC patients increased as hepatic fibrosis progressed, but serum levels of adipocytokines were not associated with hepatic fibrosis. In contrast, serum levels of AIM may not be correlated with IR as assessed by HOMA-IR, but adipocytokines were associated with IR and insulin sensitivity, and serum levels of leptin were associated with hepatic steatosis. These results suggest that serum AIM levels are not only associated with HCV-related hepatic fibrosis, but that AIM and adipocytokines are also possibly associated with pathological changes in patients with CHC via a different mechanism.

## Competing interests

HT holds endowed faculty positions in research for HGF tissue repair and regenerative medicine and has received funds from Eisai Co., Ltd.

## Authors’ contributions

KM and HU: principal investigator, data collection, subject evaluation and manuscript preparation. SM, AI, YY and TN: subject evaluation and statistical analysis. KO and KT: subject recruitment and subject evaluation. KK, TT, AM, MO, YS, MH and SE: subject recruitment and data collection. HU and HT: guarantor of the article. All authors read and approved the final manuscript.

## Pre-publication history

The pre-publication history for this paper can be accessed here:

http://www.biomedcentral.com/1471-230X/14/27/prepub
